# Neovascular Glaucoma Progress and Impact of Therapeutic Intervention in Saudi Arabia

**DOI:** 10.7759/cureus.17696

**Published:** 2021-09-03

**Authors:** Khalid AlRubaie, Abdullah Albahlal, Tariq Alzahim, Deepak P Edward, Igor Kozak, Rajiv B Khandekar

**Affiliations:** 1 Vitreoretinal Surgery, King Khaled Eye Specialist Hospital, Riyadh, SAU; 2 General Ophthalmology, King Khaled Eye Specialist Hospital, Riyadh, SAU; 3 Ophthalmology and Visual Sciences, University of Illinois College of Medicine, Chicago, USA; 4 Retina, Moorfields Eye Hospital, Abu Dhabi, ARE; 5 Ophthalmology, Faculty of Medicine, University of British Columbia, Vancouver, CAN; 6 Epidemiology and Public Health, King Khaled Eye Specialist Hospital, Riyadh, SAU

**Keywords:** neovascular glaucoma, intraocular pressure, panretinal photocoagulation, intravitreal bevacizumab, cryotherapy, pars plana vitrectomy, cyclophotocoagulation, glaucoma surgery

## Abstract

Purpose: This study aimed to present the outcomes of the therapeutic interventions for neovascular glaucoma (NVG) between 2002 and 2012 at a tertiary eye hospital in Saudi Arabia.

Methods: A retrospective chart review of the patients with NVG treated in the last 10 years at King Khaled Eye Specialist Hospital was carried out. The demographics, visual acuity, and intraocular pressure (IOP) at the baseline were compared to that, at last, follow-up. The clinical course of treated eyes and causes for poor vision were reviewed.

Results: Among 597 eyes with NVG, the mean IOP at presentation was 32 mmHg. A total of 335 eyes (56.1%) were treated with pan-retinal photocoagulation (PRP). In 459 (77%) eyes, IOP was controlled with medications or different surgeries. The vision on the last follow-up was 20/20 to 20/40 in 19 (3%) eyes, 20/50 to 20/200 in 67 (11%) eyes, <20/200 to 20/400 in 267 (45%) eyes, and <20/400 in 225 (38%) eyes. Nineteen eyes were soft/enucleated. In 45 (8%, 95% CI 6-10) eyes vision improved. The IOP was reduced to <22 mmHg in 369 (62%, 95% CI 58.2-65.9) eyes, 23-30 mmHg in 69 (12%) eyes and was > 31 mmHg in 102 (17%) eyes. In 26 (3.7%) eyes, ocular hypotony was noted. The causes of poor vision included retinal ischemia (n=75, 13%), optic nerve head cupping (n=104, 17%), retinal detachment (n= 42, 7%) and other (n=17, 3%).

Conclusion: The NVG is a serious ocular ischemic complication. Prompt therapy maintained or improved the vision and controlled IOP in 50% and more cases.

## Introduction

Neovascular glaucoma (NVG) is a serious ocular condition that often results in devastating vision loss. The key factor in treatment is the control of high intraocular pressure (IOP), which is a leading cause of permanent and substantial visual loss [1]. The pathogenesis of NVG has been linked to a locally produced angiogenic vascular endothelial growth factor (VEGF) leading to the neovascularization of the iris (NVI) and the angle (NVA) with impaired aqueous outflow [2,3]. The treatment strategies, therefore, address both increased VEGF and IOP.

Traditionally, anterior segment neovascularization has been treated promptly with panretinal laser photocoagulation or cryotherapy [4-6]. Following approval of intravitreal anti-VEGF agents, these have become an important adjunct treatment for NVG, especially in acute stages before the long-lasting effect of destructive retinal procedures takes effect [7-9]. Intraocular glaucoma surgery is warranted in advanced stages or cases non-responsive to medical and laser therapy [10,11]. The treatment outcomes in NVG can be variable.

A previous study from our hospital looked into the etiology of NVG in Saudi Arabia highlighting the role of proliferative diabetic retinopathy and retinovascular occlusions [12]. A recent epidemiologic 10-year report has demonstrated a decline in the annual incidence of NVG following the introduction of bevacizumab in the hospital’s pharmacy. Currently, there are no longitudinal reports on both medical and surgical therapeutic interventions in NVG. To fill this gap, this study presents the outcomes of therapeutic interventions for NVG at a tertiary referral eye hospital in Saudi Arabia over a 10-year period.

This article was previously presented at the ARVO 2015 Annual Meeting Abstracts: Al Rubaie K. Neovascular glaucoma (NVG) progress and impact of therapeutic intervention at King Khaled Eye Specialist Hospital, Saudi Arabia. ARVO Journals. June 2015, Vol. 56: p. 5718. (https://iovs.arvojournals.org/article.aspx?articleid=2335780) 

## Materials and methods

A retrospective chart review was performed evaluating the eyes of patients with a diagnosis of NVG at King Khaled Eye Specialist Hospital (KKESH), Riyadh, Saudi Arabia (from January 2002 to December 2012). The data were collected from all glaucoma and retina clinics during this period. The institutional review board approval for this project was obtained and the study adhered to the tenets of the Declaration of Helsinki.

The data collected during chart review included baseline characteristics, best-corrected visual acuity (BCVA; Snellen acuity); intraocular pressure (IOP) using an applanation tonometer and/or pneumo-tonometry; slit-lamp examination of the anterior segment of the eye; and retinal and optic disc examination at the baseline and last follow-up (at least after one year of management). The treatment procedures were carefully recorded for each patient. Elevated IOP was defined as an IOP of ≥21 mmHg. At the last follow-up visit recorded in the chart, visual acuity, and IOP measurements were noted. The eyes with irregular follow-up were excluded from the study. The treatment success was defined as a reduction of IOP below 22 mmHg with no antiglaucoma medications or a reduced number of antiglaucoma medications compared to baseline.

The statistical analysis was performed using SPSS software version 25 for Windows (New York, NY: IBM Corp.). For qualitative variables, we calculated frequencies and percentage proportions. To compare outcome variables among two independent variables, we calculated the odds ratio and its 95% confidence interval and two-sided "p" values. For more than two variables, we used chi-square values, degree of freedom, and two-sided "p" values. If the outcome variable was of a quantitative type, we plotted their distribution. If it was normal, we calculated the mean and standard deviation. To compare such outcome variables in two independent variables, we calculated the difference of mean, its 95% confidence interval, and two-sided "p" value.

## Results

After exclusion, we enrolled 597 eyes of 459 patients. There were 278 (61%) males and 181 (39%) females in the study. The right eye was involved in 300 (50%) eyes and the left eye was involved in the remaining 297 (50%) eyes. The mean age at presentation was 60.1 standard deviation of 13 years. The most common systemic co-morbidity was diabetes in 524 (87.8%) followed by hypertension in 255 (42.7%) eyes and hypercholesterolemia in nine (1.5%) eyes. The involvement was mainly unilateral in 455 (76.2%) eyes. Bilateral involvement was seen in 112 (18.8%) patients.

At presentation, the main visual acuity was ranging from less than 20/200 to 20/400 in 456 (76.4%) eyes. There were 438 (73.4%) eyes with an IOP of 31 mmHg or more. The majority of the affected eyes were phakic (340 {57.0%}). There were 233 (39.0%) pseudophakic eyes. Most of the affected eyes showed pre-existing optic nerve damage with 0.6-0.8 cup to disc ratio in 113 (20.0%) eyes, more than 0.8 in 55 (9.2%) eyes, and total cupping in 31 (5.2%) eyes. A total of 104 (17.4%) eyes had healthy optic nerves at the presentation. The anterior chamber angle was open (grades 3 and 4) in 176 (29.5%) eyes and a synechial closure (grades 1 and 2) was present in 264 (44.2%) eyes. The angle status was undetermined in 157 (26.3%) eyes due to poor visualization. The cornea was clear in 313 (52.4%) eyes and edematous in 260 (43.6%) eyes. Two hundred and four (34.2%) eyes presented with hyphema.

The most common cause of NVG was diabetic retinopathy (467 {78.2%} eyes) followed by central retinal vein occlusion (76 {12.7%} eyes). Table 1 below shows the causes leading to NVG.

**Table 1:** Cases of neovascular glaucoma NVG: neovascular glaucoma

Causes of NVG	Number	%
Diabetic retinopathy	467	78.2
Central retinal vein occlusion	76	12.7
Branch retinal vein occlusion	1	0.2
Central retinal artery occlusion	8	1.3
Uveitis	5	0.8
Chronic retinal detachment	3	0.5
Trauma	7	1.2
Combination of above	8	1.3
Other	21	3.5

A past history of treatment with panretinal photocoagulation (PRP) before presentation to KKESH was noted in 200 (33.5%) eyes with 124 (20.8%) eyes treated with conventional laser and 76 (12.7%) eyes with pattern scanning laser. After the presentation, the treatment of neovascularization included pattern scanning laser in 178 (29.8%) eyes, conventional laser in 157 (26.3%) eyes, intravitreal bevacizumab alone in 67 (11.2%) eyes, and retinal cryotherapy in nine (1.5%) eyes. A combination of different therapies, most often retinal laser and intravitreal bevacizumab, was recorded in 33 (5.5%) eyes.

Initially, 459 (76.9%) eyes with NVG were treated medically. The initial surgical intervention included trabeculectomy with mitomycin C in five (0.9%) eyes, glaucoma drainage device in six (1%) eyes, and diode cyclo-photocoagulation in 28 (4.7%) eyes. A combination of surgery and laser was seen in 61 (10.2%) eyes.

The success of this initial glaucoma treatment was seen in 217 (36.3%, 95% CI 32.4-40.2) eyes. During the follow-up, 331 (55.4%) eyes did not need any additional procedure. The need for additional procedures was noted in 266 (44.6%) eyes; with one procedure in 234 (39.4%) eyes, two procedures (glaucoma surgery + laser) in 29 (4.9%) eyes ,and three procedures (glaucoma surgery + laser + cryotherapy) or more (glaucoma surgery + laser + cryotherapy + bevacizumab) in three (0.6%) eyes. At the last visit, antiglaucoma medication was noted to be discontinued in 154 (25.8%) eyes. The continued medications were mainly beta-blockers in 30 (5.1%) eyes followed by brimonidine in 28 (4.7%) eyes, prostaglandin A inhibitors in eight (1.3%) eyes, and carbonic anhydrase inhibitors in seven (1.2%) eyes. A combination of the above medications was noted in 272 (45.6%) eyes.

The IOP reduction at the last follow-up was <22 mmHg in 369 (62%, 95% CI 58.1-65.9) eyes, remained 23-30 mmHg in 69 (12%) eyes, and was more than 31 mmHg in 102 (17%) eyes. In 26 (3.7%) eyes, ocular hypotony was noted. In 38 (5.8%) eyes, IOP information on the last follow-up was missing. The visual acuity on the last follow-up was 20/20 to 20/40 in 19 (3%) eyes, 20/50 to 20/200 in 67 (11%) eyes, <20/200 to 20/400 in 267 (45%) eyes, and <20/400 in 225 (38%) eyes (Table 2). In 19 (3%) eyes, the vision was either no light perception (soft eye) or could not be noted (enucleated eye).

**Table 2:** The ocular characteristics of eyes with neovascular glaucoma at the presentation IOP: intraocular pressure

		Number	Percentage proportion
Distance visual acuity	20/20 to 20/50	14	2.3%
	20/60 to 20/200	79	13.2%
	<20/200 to 20/400	456	76.4%
	<20/400 to the perception of light	45	7.5%
	No perception of light or absent eyeball	3	0.5%
History of treatment of neovascular glaucoma	Trabeculectomy +/- MMC	170	28.8%
	Valve implant surgery	29	4.9%
	Cyclo-photocoagulation	38	6.4%
	Medical treatment	178	29.8%
	No glaucoma treatment	2	0.3%
	Missing	151	25.2%
IOP (mmHg)	10-22	46	7.7%
	23-30	108	18.0%
	31 and more	438	73.3%
	Not possible	5	1%
Angle of anterior chamber	Open	176	29.5%
	Closed	264	44.2%
	Missing	157	26.3%
Lens status	Pseudophakia	233	39%
	Aphakia	14	2.3%
	Phakic eye	350	59%
Hyphema/vitreous hemorrhage	Yes	204	34.2%
	No	393	65.8%
Cup to disc ratio	Less than 0.5	104	17.4%
	0.6-0.8	112	18.8%
	>0.8	55	9.2%
	Total cupping	31	5.2%
	Missing	294	49.2%

**Figure 1:**
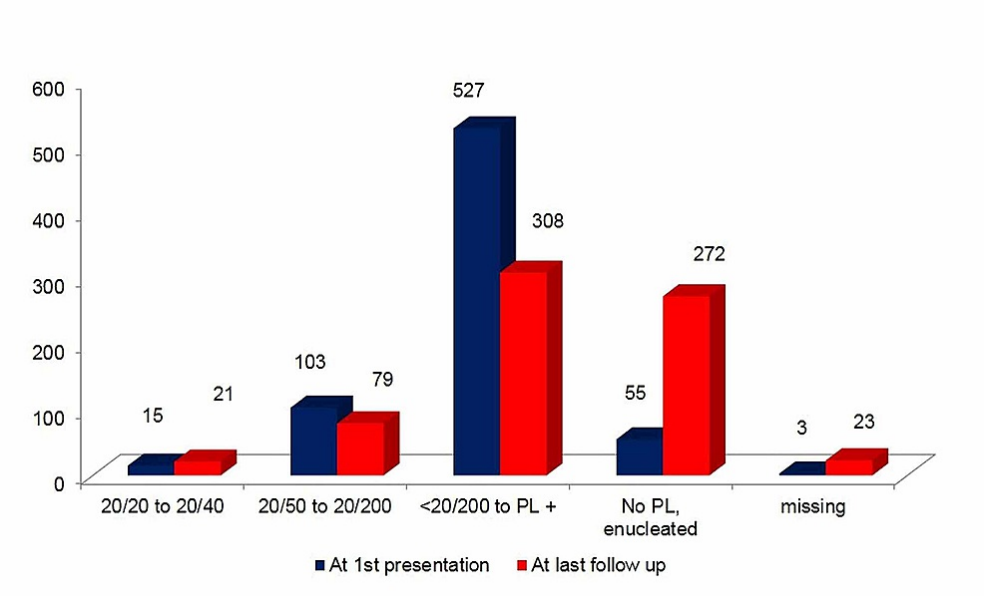
The distance visual acuity before and after the management of eye with neovascular glaucoma at King Khaled Eye Specialist Hospital The x-axis shows different visual impairment grades, the y-axis shows the number of eyes with neovascular glaucoma, the blue bar shows status at presentation, and the red bar shows status at the last follow-up.

The visual acuity had changed after the management of NVG. In 45 (8%, 95% CI 5.8-10.2) eyes the vision was improved, 305 (51%) eyes maintained the visual acuity (VA), and in 229 (38%) eyes the vision got worse (Figure 1).

A severe visual loss (VA < 20/200) was noticed in 83% of the cases despite the treatment (<20/200 to 20/400 in 267 {45%} eyes and <20/400 in 225 {38%} eyes). The main causes of poor vision at the last visit included retinal ischemia (75 eyes {13%}), optic nerve head cupping (104 eyes {17%}), retinal detachment (42 eyes {7%}), and other causes (17 eyes {3%}). At presentation, NVG was bilateral in 112 (19%) of the cases. At the last follow-up, NVG was noted in 174 (29.1%) fellow eyes. Thus, 62 (10.4%) eyes had new NVG that was not diagnosed in fellow eye at the presentation. 

## Discussion

Neovascular glaucoma is an ischemic ocular disease that leads to elevated IOP. The management of NVG can be divided into two categories: (1) the management of the underlying disease and (2) the management of high IOP when it develops [1]. The conditions leading to ischemic retinopathy, such as retinovascular occlusions or diabetic retinopathy, are traditionally the most frequent underlying disease in NVG [1,13]. Complicated diabetic retinopathy was the major cause of NVG in our series affecting more than three-quarters of the cases. Retinovascular occlusions were the second most common underlying pathology. Both are likely to be related to the recent epidemic of diabetes and hypertension in the Middle East with a higher proportion of uncontrolled diabetic retinopathy in the Saudi population [14,15].

Retinal laser photocoagulation was the main treatment for the underlying disease(s) in patients of this cohort. Traditionally, retinal ablation by laser and/or cryotherapy has been the first line of treatment for NVG [16]. There is strong evidence that panretinal photocoagulation is the treatment of choice for the prevention of the development of NVG if retinal ischemia is a factor - level A recommendation by the American Academy of Ophthalmology [17]. Intravitreal bevacizumab alone and in the combination with retinal laser was administered to 11.2% and 5.5% of the patients in the study, respectively. These predominantly included advanced cases. The role of antiangiogenic agents in the treatment of the ischemic components of NVG has been established [8,18,19]. It has been shown to induce rapid resolution of iris and iridocorneal angle vessels as demonstrated angiographically. The effect has been observed after both intravitreal and intracameral administration [18-20]. The majority of our patients received intravitreal injections. While PRP provides a more permanent reduction of the ischemic angiogenic stimulus, bevacizumab-induced regression of iris neovascularization is often temporary and recurrence is possible [21,22].

The management of high IOP in NVG usually starts with medical therapy which was also the case in this cohort. Topical therapy can prevent visual loss and relieve associated pain or discomfort. IOP is lowered invariably by means of various aqueous suppressants (beta-blockers, alpha-adrenergic, and carbonic anhydrase inhibitors). The prostaglandins may not be of much help because they work by increasing the uveal outflow, which may be covered by a neovascular membrane that forms at the angle [1]. The medical therapy in our series was initially used for 77% of patients. The combination of the above-mentioned agents had achieved IOP control in 36% of all patients at the last visit.

Surgical intervention is indicated when medical therapy is inadequate to control IOP, particularly if a synechial angle closure from NVA has occurred [19]. Cyclophotocoagulation (CPC) was the most commonly performed initial surgical intervention in our series (4.7%). The disadvantage of this surgical option is that it is often difficult to titrate the effect of CPC on the ciliary body and frequently more than one treatment is necessary to attain effective IOP control [23]. Excessive laser treatment can lead to hypotony and phthisis [19].

The most commonly used filtering procedure for NVG is trabeculectomy combined with or without mitomycin C or 5-fluorouracil [24]. The trabeculectomy with mitomycin C was performed in 0.9% of patients as an initial treatment. This kind of procedure for NVG is moderately successful in the long term [19]. Its reported success rate is 62.6% in one year, declining to 51.7% by five years of follow-up [25]. Similarly, in our study, this procedure was supplemented with an additional procedure or medical therapy. Glaucoma drainage devices have been considered as an option in the management of NVG where there is a high risk of failure from conventional filtering surgery [1]. Their success is less dependent on control of intraocular inflammation and the failure of a filtering bleb [26]. These procedures were performed on 1% of our cases at the presentation.

The control in IOP (defined as IOP < 22 mmHg) has improved from 7.7% at presentation to 62% at the last visit. The success of initial glaucoma treatment was seen in 36.3% of patients with a need for additional surgical procedures in 44.5%. A recent study by Sasamoto et al. reported the need for antiglaucoma surgery in 43% of patients who had prior procedures such as PRP, vitrectomy surgery, glaucoma medication, and intravitreal bevacizumab. They have also demonstrated that the logarithmically transformed aqueous VEGF levels were highly significantly correlated with IOP levels [27]. In a retrospective case series by Kotecha et al., 41% of eyes required glaucoma surgery in spite of initial success with bevacizumab alone [28]. Bevacizumab-induced regression of NVI and NVA may stabilize elevated IOP via a reduction of fibrovascular contraction angle closure and reducing the need for surgical IOP-lowering interventions [29]. However, many of the patients in our cohort had or developed advanced, refractory NVG with a significant angle-closure requiring incisional surgical intervention or cyclodestructive procedure to reduce elevated IOP.

The visual acuity in our patients improved in general even though the majority of them had a severe visual loss (<20/200). Therapy of NVG has been associated with improvements in visual acuity in other studies [25-29]. Optic nerve damage was the most common cause in identifiable cases indicating the need for urgent control of IOP in such cases. Other causes of decreased vision included retinal ischemia, chronic detachment, macular edema, and media opacity.

An obvious limitation of this study is a retrospective data analysis. While attention was paid to include only clean data, standardizations of some observations are impossible. For example, the effect of a particular surgical procedure or medical therapy cannot be evaluated rather than the effect of the overall therapy or combination of procedures. The strength of the study is robust, longitudinal data from the tertiary eye care center.

A high prevalence of diabetes and diabetic retinopathy among the Middle Eastern population should be noted as the risk of neovascular glaucoma is also high [30,31]. The management with Ahmed valve for refractory glaucoma including NVG is not promising in Arab patients [32]. This matches with the outcomes of the present study. Therefore, the caregiver should explain to the patients and relatives about the guarded prognosis of surgical management of NVG.

## Conclusions

Neovascular glaucoma is a serious complication of ocular ischemic syndromes that occurs frequently in patients with prevalent underlying diseases and susceptible populations such as in the Middle East. A prompt therapy targeting ischemia included ablation of ischemic tissue and antiangiogenic therapy with a combination of glaucoma medical and surgical therapy maintained or improved vision and controlled IOP in more than half of our patients.
